# Evaluating the quality of life of cancer patients: assessments by patients, significant others, physicians and nurses

**DOI:** 10.1038/sj.bjc.6690655

**Published:** 1999-09

**Authors:** K C A Sneeuw, N K Aaronson, M A G Sprangers, S B Detmar, L D V Wever, J H Schornagel

**Affiliations:** Division of Psychosocial Research and Epidemiology, the Netherlands Cancer Institute/Antoni van Leeuwenhoek Hospital, Plesmanlaan 121, Amsterdam, 1066 CX, The Netherlands; Department of Internal Medicine, the Netherlands Cancer Institute/Antoni van Leeuwenhoek Hospital, Amsterdam, The Netherlands; Department of Medical Psychology, Academic Medical Center, Amsterdam, The Netherlands

**Keywords:** quality of life, proxy respondents, agreement, questionnaire

## Abstract

This study examined the usefulness of caregiver ratings of cancer patients' quality of life (QOL), an issue of relevance to both adequate patient care and to the possible use of proxy QOL raters in clinical studies. We compared QOL ratings of 90 cancer patients receiving inpatient chemotherapy with those provided by their significant others (most often the spouse), physicians and nurses. During patients' scheduled appointment for receiving chemotherapy on a clinical ward, all raters completed independently the Dartmouth COOP Functional Health Assessment charts/WONCA, an instrument developed by a cooperative group of primary care physicians to briefly assess a core set of seven QOL domains (physical fitness, feelings, daily and social activities, overall health, pain and quality of life) by single items with five response options. With few exceptions, mean scores of the proxy raters were equivalent or similar to those of the patients. Most patient–proxy correlations varied between 0.40 and 0.60, indicating a moderate level of agreement at the individual level. Of all comparisons made, 41% were in exact agreement and 43% agreed within one response category, leaving 17% more profound patient–proxy discrepancies. Disagreement was not dependent on the type of proxy rater, or on raters' background characteristics, but was influenced by the QOL dimension under consideration and the clinical status of the patient. Better patient–proxy agreement was observed for more concrete questions (daily activities, pain) and for patients with either a very good (ECOG 0) or poor (ECOG 3) performance status. The results indicate that both significant others and health care providers can be useful sources of information about cancer patients' QOL. © 1999 Cancer Research Campaign


*British Journal of Cancer* (1999) **81**(1), 87–94
© 1999 Cancer Research Campaign
Article no. bjoc.1999.0655

### Evaluating the quality of life of cancer patients: assessments by patients, significant others, physicians and nurses

**KCA Sneeuw[1], NK Aaronson[1], MAG Sprangers[3], SB Detmar[1], LDV Wever[1] and JH Schornagel[2]**

[1]Division of Psychosocial Research and Epidemiology, the Netherlands Cancer Institute/Antoni van Leeuwenhoek Hospital, Plesmanlaan 121, 1066 CX Amsterdam, The Netherlands; [2]Department of Internal Medicine, the Netherlands Cancer Institute/Antoni van Leeuwenhoek Hospital, Amsterdam, The Netherlands; [3]Department of Medical Psychology, Academic Medical Centre, Amsterdam, The Netherlands

**Summary** This study examined the usefulness of caregiver ratings of cancer patients' quality of life (QOL), an issue of relevance to both adequate patient care and to the possible use of proxy QOL raters in clinical studies. We compared QOL ratings of 90 cancer patients receiving inpatient chemotherapy with those provided by their significant others (most often the spouse), physicians and nurses. During patients' scheduled appointment for receiving chemotherapy on a clinical ward, all raters completed independently the Dartmouth COOP Functional Health Assessment charts/WONCA, an instrument developed by a cooperative group of primary care physicians to briefly assess a core set of seven QOL domains (physical fitness, feelings, daily and social activities, overall health, pain and quality of life) by single items with five response options. With few exceptions, mean scores of the proxy raters were equivalent or similar to those of the patients. Most patient–proxy correlations varied between 0.40 and 0.60, indicating a moderate level of agreement at the individual level. Of all comparisons made, 41% were in exact agreement and 43% agreed within one response category, leaving 17% more profound patient–proxy discrepancies. Disagreement was not dependent on the type of proxy rater or raters' background characteristics, but was influenced by the QOL dimension under consideration and the clinical status of the patient. Better patient–proxy agreement was observed for more concrete questions (daily activities, pain) and for patients with either a very good (ECOG 0) or poor (ECOG 3) performance status. The results indicate that both significant others and health care providers can be useful sources of information about cancer patients' QOL.

**Keywords:** quality of life; proxy respondents; agreement; questionnaire

Quality of life (QOL) assessment is increasingly being used in clinical cancer research as an important outcome for assessing treatment effects (Osoba, 1994; Medical Research Council Lung Cancer Working Party, 1996). Additionally, recent attention has been directed toward the possibility of employing individual QOL assessments in daily clinical practice (Wasson et al, 1992; Detmar and Aaronson, 1998). Both efforts are aimed at the factoring of quality of life considerations explicitly into the medical decision-making process. Given that the patient is the most appropriate source of information on his or her QOL, such assessments are primarily derived from the patients themselves. Yet, there are several reasons for studying the value of proxy QOL ratings provided by the patients' caregivers at home (e.g. spouses, other family members or friends) and in the clinic (e.g. physicians and nurses).

First, it is important to know the extent to which caregivers can assess accurately a patient's level of functioning and well-being, in that such assessments can influence significantly decisions regarding treatment and patient care (Ford et al, 1994; Schor et al, 1995; Macquart-Moulin et al, 1997). Second, there are a number of research situations in which the patient may not be able or willing to provide QOL ratings. Problems with self-report may arise when patients have cognitive impairments or communication deficits, when they experience severe symptom distress, or when an interview is physically or emotionally too burdensome. For such patients, caregivers might be employed as complementary or alternative sources of information on their QOL (Magaziner, 1[illegible]; Sprangers and Aaronson, 1992).

Historically, physicians and nurses have played a central role in evaluating patients' QOL, albeit in the limited sense of providing ratings of performance status, treatment toxicity and pain intensity. In earlier work (Sprangers and Aaronson, 1992), we identified 35 published reports evaluating QOL in patients with chronic disease in which ratings from health care providers and patients were compared. These studies indicated that the concordance between patients' and caregivers' QOL ratings was far from optimal, but also suggested a clear need for more methodologically sound studies using larger sample sizes and standard QOL questionnaires. Recently, two studies among large samples of cancer patients have shown more promising results. Stephens et al (1997) reported high levels of agreement between patients' and physicians' ratings on a range of key physical symptoms of the Rotterdam Symptom Checklist as assessed in two randomized trials of palliative treatment for patients with lung cancer. Importantly, they found that the conclusions based on the between-treatment comparisons for these symptoms were essentially the same whether one used the physicians' or the patients' QOL ratings. Sneeuw et al (1997a) also provided encouraging findings

*Received 24 August 1998*
*Revised 29 January 1999*
*Accepted 12 April 1999*

*Correspondence to:* NK Aaronson on the validity of physicians' ratings of several general aspects of cancer patients' QOL as measured by the COOP/WONCA charts, an instrument developed by a cooperative group of primary care physicians to briefly assess a core set of QOL domains. Relative to the patients, the physicians were more efficient in detecting changes over time in physical fitness and overall health, but less so in relation to social function and pain.

Increasingly, attention has been paid to the potential use of significant others, particularly spouses, other relatives or friends taking care of the patient in the home situation, as raters of cancer patients' quality of life (Grassi et al, 1996; Kurtz et al, 1996; Sneeuw et al, 1997*a*, 1997*b*, 1998). Theoretically, significant others would seem to be a better choice as proxy raters of patients' QOL than health care providers. They have the opportunity to observe the patient engaging in a wide range of activities over extended periods of time and may have better access to the patient's thoughts and feelings than do health care professionals (Aaronson, 1991). This position has been supported by a number of small studies among cancer patients (Slevin et al, 1988; Blazeby et al, 1995; Sigurdardottir et al, 1996), showing slightly elevated levels of patient–proxy agreement for significant others as compared to either physicians or nurses. On the other hand, as we suggested earlier (Sprangers and Aaronson, 1992), ratings provided by significant others can also be biased by the caregiving function of the rater. In line with this suggestion, other studies have reported that significant others and health care providers evaluate patients' QOL with a comparable degree of (in)accuracy (Grassi et al, 1996; Sneeuw et al, 1997*a*).

The purpose of the current study was to examine the usefulness of caregiver ratings of the QOL of a heterogeneous sample of cancer patients by assessing the level of agreement between patient and proxy responses to a brief standardized QOL instrument. This study contributes to the growing body of research on the value of proxy QOL ratings by providing a head-to-head comparison of the levels of patient–significant other, patient–physician and patient–nurse agreement. Secondly, we investigated the relative effects of the type of proxy rater, the type of question/QOL domain, the patients' clinical status, and several sociodemographic characteristics of all raters on the level of patient–proxy agreement. Finally, in addition to the usual pairwise comparisons, the availability of responses from four different sources also allowed for comparisons of four ratings simultaneously.

### METHODS

### Study sample

The current analysis was based on data obtained from participants in a larger study examining the value and limitations of proxy ratings of cancer patients' QOL (Sneeuw et al, 1997*a*, 1998). The total patient sample was composed of patients with a range of cancer diagnoses who attended the Netherlands Cancer Institute/Antoni van Leeuwenhoek Hospital for treatment involving either inpatient or outpatient chemotherapy. For all patients, QOL ratings were obtained from their significant others and treating physicians. Nurse ratings were obtained for hospitalized patients only, given the more frequent and consistent nurse–patient contact in the inpatient clinic setting. Since the aim of the current analysis was to provide a head-to-head comparison of significant others, physicians and nurses, we focused on the patients who were recruited from one of the inpatient wards. Exclusion criteria included participation in a concurrent QOL study, age less than 18 years and a lack of basic proficiency in Dutch.

Eligible patients received a full, verbal and written explanation of the purpose and procedures of the study. Consenting patients were requested to identify a significant other (i.e. spouse or other close companion) and to ask them to participate in the study. The significant others were also provided with verbal and written information on the study. Given their central role in the treatment and care of patients receiving inpatient chemotherapy, all ward physicians (interns and residents) and nurses who worked at the specific inpatient ward over the entire study period were asked to take part in the study. The physicians and nurses also received a full explanation of the purposes and procedures of the study.

### Measures and procedures

Quality of life was assessed by means of the Dartmouth COOP Functional Health Assessment charts/WONCA (Scholten and Van Weel, 1992; Van Weel, 1993; Van Weel et al, 1995). The COOP/WONCA charts are an adapted version of the Dartmouth COOP charts (Nelson et al, 1987), developed by a cooperative group of primary care physicians. The reliability and validity of the original COOP charts have been established in a number of studies (Nelson et al, 1987, 1990). While psychometric testing of the revised version is ongoing, there is ample evidence that the COOP/WONCA charts used here also yield reliable and valid data (Scholten and Van Weel, 1992; Van Weel et al, 1995). The COOP/WONCA charts assess QOL at a generic level, covering a core set of domains, including physical fitness, feelings, daily and social activities, overall health and pain. An additional chart assessing overall QOL was also included. Each chart consists of a descriptive title, a question referring to a single aspect of the patient's QOL in the past 2 weeks, and five response categories illustrated by drawings. Scores range from 1 to 5, with 1 representing the best and 5 indicating the worst level of functioning or well-being (Figure 1).

Patients, significant others, physicians and nurses were asked to complete the COOP/WONCA charts independently of each other. The proxy questions were identical to those of the patients, but were rephrased slightly so that each question referred to the patient. Also, standard instructions were provided in which proxies were asked to try to view the situation from the perspective of the patient, and to complete the questionnaire as they thought the patient would. While patients and significant others received each question on a separate sheet, for practical reasons, the seven questions were concentrated on a single form for use by the physicians and nurses.

Data were collected by self-administration during patients' scheduled clinical ward stay at which they received a second cycle of chemotherapy. A research assistant was present to check for missing data. In most cases, the significant other completed the questionnaire while visiting the patient at the clinical ward. In these cases, the significant other was asked to fill out the questionnaire in a separate room in the presence of another research assistant. When the significant other could not be approached at the hospital, an arrangement was made to have the questionnaire completed at home and returned in a self-addressed, stamped envelope. The physicians and nurses on the clinical ward were asked to complete the COOP/WONCA charts in their office, on the same day as did their patients.

© 1999 Cancer Research Campaign

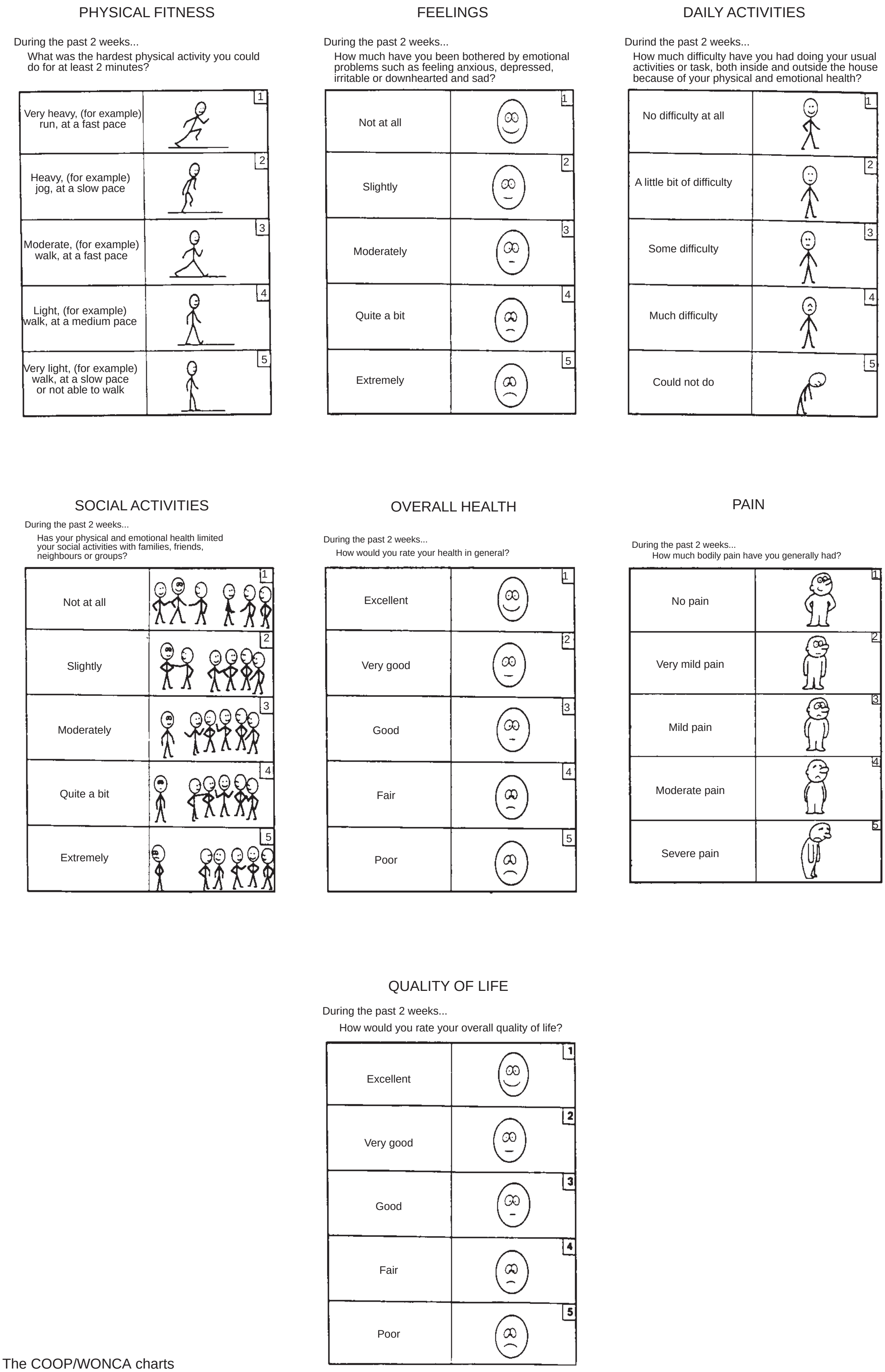

**Figure 1** The COOP/WONCA charts

The research assistants also rated the performance status of the patients, using the Eastern Cooperative Oncology Group (ECOG) performance status scale (Zubrod et al, 1960; Sorensen et al, 1993). The ECOG scale describes the patients' level of functioning in terms of activity, ambulatory status and need for care. An ECOG score of 0 means normal activity, ECOG 1 means some restriction in activity but ambulatory, ECOG 2 means capable of self-care but some daytime spent in bed, ECOG 3 means more than 50% of daytime in bed, and ECOG 4 means completely bedridden. To establish the patients' performance status, the research assistants used a standard set of questions based on guidelines recommended by Schag et al (1984).

### Data analysis

The level of agreement between patient and proxy ratings was examined in several ways. Mean scores of patients and proxies were compared to examine agreement at the group level. Statistically significant differences in mean scores, as indicated by paired Student's *t*-tests, were interpreted as providing evidence of systematic differences between raters (Lee et al, 1989; Marshall et al, 1994). To interpret the size of observed differences, the mean difference scores were standardized by relating these scores to their standard deviations. Given the similarity to effect size ($d$) calculations for paired observations (Cohen, 1988), a standardized difference of $d = 0.2$ was taken to indicate a small difference, $d = 0.5$ a moderate difference, and $d = 0.8$ a large difference.

The intraclass correlation (ICC) coefficient was used as an indicator of chance-corrected agreement between patient and proxy ratings at the individual level (Bartko, 1966; Lee et al, 1989). Guidelines for the ICC as a measure of the strength of agreement were labelled as follows: ≤0.40, poor to fair agreement; 0.41–0.60, moderate agreement; 0.61–0.80, good agreement; 0.81–1.00, excellent agreement (Landis and Koch, 1977). For ordinal data, as used here, the ICC coefficient has been demonstrated to be mathematically equivalent to the weighted kappa statistic (Fleiss and Cohen, 1973).

Additionally, response agreement between patient and proxy raters was assessed by calculating the proportions of exact agreement, adjacent-category differences, and differences of more than one response category. The latter, being interpreted as large patient–proxy discrepancies, were used to investigate further the relative effects of the type of question (the seven COOP/WONCA charts), the type of proxy rater (significant other, physician, nurse), the patients' performance status (ECOG score), and sociodemographic characteristics (age, sex, education) of all raters on the level of patient–proxy agreement.

Finally, simultaneous comparisons of the four raters were made, establishing the proportions of complete agreement and deviant scores. Complete (or nearly complete) agreement was defined as those cases in which all four raters agreed within one response category (e.g. 2,2,3,2). A deviant score was defined as the situation in which only one of the raters differed more than one response category from the remaining three raters (e.g. 1,4,4,4).

### RESULTS

### Sample characteristics

Of 115 eligible patients, 100 agreed to participate in the study (87% response rate). Seven of the 15 non-respondents chose not to participate in the study due to very poor physical or emotional condition. The remaining eight non-respondents did not provide any specific reason for not participating other than a general lack of interest in the study. Significant other, physician and nurse COOP/WONCA ratings were obtained for 93, 97 and 99 of the 100 patients respectively. The current analyses focus on the 90 patients for whom all four sources of QOL ratings were available.

Patients had a range of cancer diagnoses, with lung cancer (23%), advanced breast cancer (22%), testicular cancer (16%) and soft tissue sarcoma (14%) being the most prevalent. The patient sample was heterogeneous in terms of performance status (eight patients ECOG 0, 54 patients ECOG 1, 21 patients ECOG 2, and seven patients ECOG 3). The patient sample included 48 men (53%) and 42 women (47%). Patients' age ranged from 19 to 75 years, with a mean age of 49 years.

The significant others were most often the patients' spouse or partner (76%). The remaining significant others were parents (7%), adult children (6%), other relatives (7%), or friends (4%). Most of them were living in the same household as the patients (82%). Fifty-one significant others were women (57%) and 39 men (43%). Their mean age was 49 years, with a range of 20–78 years.

Fifteen ward physicians (ten female, five male) participated in the study. The physicians were 26–36 years of age (mean age 30 years) and had, on average, 25 months (range 2–60 months) of work experience as a physician. The nurse sample consisted of 35 nurses (29 female, six male). The nurses varied in age from 25 to 56 years (mean age 32 years). On average, they had 11 years (range 4–33 years) of work experience, of which 5 years (range 1–22 years) as an oncology nurse.

**Table 1** Comparison of patient and proxy mean scores on the COOP/WONCA charts

| | Patient Mean ± s.d. | Significant other Mean ± s.d. | Physician Mean ± s.d. | Nurse Mean ± s.d. |
|---|---|---|---|---|
| Physical fitness | 3.3 ± 1.3 | 3.5 ± 1.2 | 3.5 ± 1.0 | 3.4 ± 1.0 |
| Feelings | 2.2 ± 1.0 | 2.7 ± 1.1[a] | 2.6 ± 1.0[a] | 2.3 ± 0.9 |
| Daily activities | 3.1 ± 1.3 | 3.3 ± 1.2[a] | 3.0 ± 1.1 | 3.0 ± 1.1 |
| Social activities | 2.7 ± 1.4 | 2.7 ± 1.3 | 2.7 ± 1.2 | 2.6 ± 1.0 |
| Overall health | 3.4 ± 1.0 | 3.7 ± 0.9[a] | 3.4 ± 0.9 | 3.5 ± 0.8 |
| Pain | 2.2 ± 1.2 | 2.5 ± 1.2[a] | 1.8 ± 1.1[b] | 2.1 ± 1.1 |
| Quality of life | 3.2 ± 1.1 | 3.5 ± 0.9[a] | 3.2 ± 0.9 | 3.3 ± 0.8 |

Note. Scores range from 1 to 5 with a higher score representing a more impaired level of functioning or well-being. [a] Proxy scores significantly higher ($P < 0.05$) than patient scores. [b] Proxy scores significantly lower ($P < 0.05$) than patient scores.

### Patient–proxy agreement

Table 1 shows the mean scores of the patients and proxy respondents on the COOP/WONCA charts. For seven of the 21 patient–proxy comparisons statistically significant differences were noted, indicating systematic differences between patient and proxy ratings. The significant others rated the patients as having more impaired levels of feelings and daily activities, more pain, and poorer overall health and quality of life than did the patients themselves. The physicians' ratings indicated more impaired feelings, but less pain than those of the patients. No statistically significant differences were observed between the patients' and

© 1999 Cancer Research Campaign

**Table 2** Intraclass correlations between patient and proxy scores on the COOP/WONCA charts

| | Patient *vs* Significant other ICC | Patient *vs* Physician ICC | Patient *vs* Nurse ICC | Average ICC |
|---|---|---|---|---|
| Physical fitness | 0.57 | 0.53 | 0.38 | *0.49* |
| Feelings | 0.48 | 0.37 | 0.43 | *0.43* |
| Daily activities | 0.66 | 0.56 | 0.58 | *0.60* |
| Social activities | 0.47 | 0.20 | 0.43 | *0.37* |
| Overall health | 0.44 | 0.45 | 0.41 | *0.43* |
| Pain | 0.64 | 0.50 | 0.66 | *0.60* |
| Quality of life | 0.37 | 0.51 | 0.36 | *0.41* |
| Average | *0.52* | *0.45* | *0.46* | *0.48* |

**Table 3** Percentage of large discrepancies between patient and proxy scores on the COOP/WONCA charts

| | Patient *vs* Significant other % | Patient *vs* Physician % | Patient *vs* Nurse % | Total[a] % |
|---|---|---|---|---|
| Physical fitness | 18 | 13 | 21 | *17* |
| Feelings | 13 | 16 | 11 | *13* |
| Daily activities | 13 | 20 | 14 | *16* |
| Social activities | 27 | 38 | 25 | *30* |
| Overall health | 9 | 10 | 9 | *9* |
| Pain | 17 | 20 | 10 | *16* |
| Quality of life | 21 | 10 | 16 | *16* |
| *Total*[b] | *17* | *18* | *15* | *17*[c] |

[a]Across the three pairs of raters for 90 patients (3 × 90 = 270 comparisons).
[b]Across the seven questions for 90 patients (7 × 90 = 630 comparisons).
[c]Across the three pairs of raters and seven questions for 90 patients (3 × 7 × 90 = 1890 comparisons).

nurses' ratings. The standardized differences (effect size *d*) for the five systematic differences observed between patients and significant others ranged between 0.22 and 0.30 for daily activities, overall health and quality of life, and was 0.47 for feelings (not shown in tabular form). The standardized differences of the two systematic patient–physician differences were 0.40 for feelings and –0.40 for pain.

To examine patient–proxy agreement at the individual patient level, a 3 × 7 matrix was constructed of the intraclass correlations between the ratings of the three patient–proxy pairs on the seven COOP/WONCA charts. The average ICC over all 21 correlations was 0.48 (Table 2). Similar levels of agreement were noted between the three patient–proxy pairs, with the average ICCs ranging from 0.45 to 0.52. Average ICCs on the seven COOP/WONCA charts ranged from 0.37 for social activities to 0.60 for daily activities and pain. Relatively lower levels of agreement, as indicated by ICCs <0.40, were observed for five of the 21 patient–proxy comparisons: between patients' and significant others' ratings of overall quality of life, between patients' and physicians' ratings of feelings and social activities, and between patients' and nurses' ratings of physical fitness and overall quality of life.

### Factors affecting response agreement

Given three pairs of ratings on the seven COOP/WONCA charts for each of the 90 patients, a potential total of 1890 patient–proxy comparisons could be made. Due to missing data, 11 comparisons were not possible, leaving 1879 comparisons between patient and proxy ratings. Of these, 764 (41%) were in exact agreement, and 801 (43%) agreed within one response category. Large patient–proxy discrepancies (i.e. more than one response category of difference) were noted on 314 (17%) occasions. As shown in Table 3, the percentages of large discrepancies varied across the

**Table 4** Percentage of large discrepancies across all comparisons ($n$ = 1890)[a] broken down by explanatory variables

| Proxy characteristics | No. of comparisons | % Large discrepancies | Patient characteristics | No. of comparisons | % Large discrepancies |
|---|---|---|---|---|---|
| Type of proxy rater | | | Performance status | | |
| Significant other | 628 | 17% | ECOG 0 | 165 | 10% |
| Physician | 624 | 18% | ECOG 1 | 1128 | 16% |
| Nurse | 627 | 15% | ECOG 2 | 440 | 24% |
| | | | ECOG 3 | 146 | 10% |
| Proxies' age | | | Patients' age | | |
| ≤ 40 | 1362 | 16% | ≤ 40 | 609 | 16% |
| 41–55 | 321 | 18% | 41–55 | 623 | 19% |
| 55+ | 189 | 15% | 55+ | 647 | 15% |
| Proxies' sex | | | Patients' sex | | |
| Male | 520 | 16% | Male | 1000 | 16% |
| Female | 1359 | 17% | Female | 879 | 17% |
| Proxies' education | | | Patients' education | | |
| Low | 203 | 19% | Low | 524 | 22% |
| Intermediate | 264 | 14% | Intermediate | 793 | 14% |
| High | 1405 | 17% | High | 562 | 16% |

[a]Across three pairs of raters and seven questions for 90 patients (3 × 7 × 90 = 1890 comparisons); number of comparisons varies due to missing data.

© 1999 Cancer Research Campaign

**Table 5** Number of occasions with one of the raters having a deviant score[a]

| | No. of Comparisons | No. of deviant scores | | | | |
|---|---|---|---|---|---|---|
| | | Patient | Significant other | Physician | Nurse | Total |
| Physical fitness | 90 | 6 | 6 | 1 | 3 | *16* |
| Feelings | 89 | 3 | 1 | 4 | 3 | *11* |
| Daily activities | 90 | 4 | 2 | 1 | 3 | *10* |
| Social activities | 85 | 4 | 5 | 7 | 2 | *18* |
| Overall health | 89 | 3 | 0 | 3 | 0 | *6* |
| Pain | 88 | 1 | 0 | 4 | 1 | *6* |
| Quality of life | 90 | 5 | 1 | 0 | 0 | *6* |
| Total | *621* | *26* | *15* | *20* | *12* | *73* |

[a]Score of one rater being more than one response category different from those of the other three raters.

seven COOP/WONCA charts. For all patient–proxy pairs, relatively few large discrepancies (on average 9%) were noted for the overall health ratings, and relatively many large discrepancies (on average 30%) for ratings of social activities. The large discrepancies were evenly distributed across the three patient–proxy pairs, indicating that disagreement was not dependent on the type of proxy rater.

Table 4 displays the effects of the type of proxy rater, the patients' performance status and sociodemographic characteristics of the patients and proxy raters on the percentages of large patient–proxy discrepancies. As was observed for the type of proxy rater, the proportions of large patient–proxy discrepancies varied within narrow margins across the different age, sex and education subgroups. For patients' performance status, however, a more substantial effect was observed. For patients with either a very good (ECOG 0) or very poor (ECOG 3) performance status, the percentage of large discrepancies was 10%. For patients with a slightly impaired (ECOG 1) or moderately impaired (ECOG 2) performance status, the corresponding figures were 16% and 24% respectively.

### Simultaneous comparison of four raters

Given the seven COOP/WONCA items and 90 patients, a potential total of 630 simultaneous comparisons of four responses could be made. Due to missing data for nine patients, 621 comparisons were possible. Complete or nearly complete agreement, defined as those cases in which the four raters agreed within one response category (e.g. 2,2,3,2), was noted on 340 occasions (55%). Deviant scores, defined as those cases in which only one of the raters differed more than one response category from the remaining three (e.g. 1,4,4,4), were found in 73 cases (12%). Table 5 shows that, of those 73 deviant scores, the patient was the deviant rater on 26 occasions, the significant other on 15 occasions, the physician on 20 occasions and the nurse on 12 occasions. Deviant scores were most often found for physical fitness and social activities (16 and 18 occasions respectively). For physical fitness, 13 of the 16 deviant ratings were in the positive direction (i.e. the deviant rater scoring 'very heavy', the other raters scoring 'moderate' to 'very light').

### DISCUSSION

This report describes a head-to-head comparison of significant others, physicians and nurses as proxy raters of the QOL of patients with cancer receiving inpatient chemotherapy, as assessed by the seven questions of the COOP/WONCA charts. To examine patient–proxy agreement at the group level, mean scores of patients and proxy respondents were compared. This is of particular importance for using proxy QOL ratings in clinical trials, where groups of patients are compared rather than individual patients. Significant others systematically reported more problems than did the patients themselves for five of the seven QOL domains. Physicians also rated more emotional problems than did the patients, but underreported pain. The latter finding confirms earlier reports of physicians' tendency to underestimate patients' pain intensity (Hodgkins et al, 1985; Grossman et al, 1991; Au et al, 1994). Interestingly, no systematic differences in mean scores were noted between patient and nurse ratings, suggesting that nurses may be the most suitable source of proxy information in clinical trials of hospitalized cancer patients.

It is important to note that the statistical significance of observed differences is, in part, dependent on sample size. Unfortunately, there are no clear-cut ways of interpreting the importance of statistically significant differences. Ideally, one would like to know at which size a systematic difference is clinically meaningful. Although attempts have been made in this direction (King, 1996; Osoba et al, 1998), the QOL literature does not yet provide unequivocal recommendations. As a 'second best' alternative for interpreting the size of systematic differences, standardized differences (or effect sizes) were employed. In view of guidelines recommended by Cohen (Cohen, 1988), the systematic differences that were observed for seven of the 21 patient–proxy comparisons were small to moderate in magnitude. The larger differences observed between the patients' ratings and those of their significant others and physicians in the area of emotional functioning suggest that the use of proxy respondents may introduce a bias in this QOL domain. Overall, however, the results indicate that only a modest degree of response bias would be introduced when substituting patients' self-report of their QOL by proxy ratings.

To examine patient–proxy agreement at the individual patient level, we employed a combination of the ICC, being a suitable statistical measure for ordinal data (Nelson et al, 1990) and more appealing measures such as the proportions of exact agreement, adjacent-category differences (which can be described as global or approximate agreement) (Sneeuw et al, 1997*a*, 1997*b*), and differences of more than one response category. With few exceptions, the patient–proxy correlations varied between 0.40 and 0.60, usually interpreted as representing a moderate level of agreement (Landis and Koch, 1977). The proportions exact and global agreement indicated that significant others, physicians and nurses provided identical or very similar ratings to those of the patients in the vast majority of cases. Larger differences (of more than one response category on a 1–5 range) between patient and proxy ratings were found in approximately 15% of the cases.

Concordance between patient and proxy ratings appears to be dependent, in part, on the QOL dimension under consideration. As has been suggested earlier (Magaziner, 1992, 1997; Sprangers and Aaronson, 1992), both the type of question and the way in which questions are asked can affect the level of patient–proxy agreement. Specifically, the visibility of the functional problem or

 © 1999 Cancer Research Campaign symptom, the concreteness of the question, as well as the number, type and content of response categories can all influence levels of patient–proxy agreement. For the COOP/WONCA charts, higher rates of agreement were therefore expected for physical fitness, daily activities and pain. For the latter two domains, patient–proxy correlations were indeed relatively high. For the physical fitness question, as will be discussed later, responses of questionable validity may have been provided on several occasions. Since problems with the validity of this question were also noted in earlier work, (Sneeuw et al, 1997a; Siu et al, 1993) we would encourage efforts to improve the content of the physical function question and/or the response options. Relatively lower levels of patient–proxy agreement were expected and found for the more private domains of emotional and social function, and the broad concepts of overall health and quality of life.

As is the case for mean scores at the group level, there are no predefined ways to interpret the level of patient–proxy agreement at the individual level. Based on general statistical guidelines (Landis and Koch, 1977), poor to fair agreement was noted for five of the 21 patient–proxy comparisons. One might conclude that such low correlations make proxy ratings unacceptable for either clinical or research use. However, as for all other patient–proxy comparisons, the proportions of exact and global agreement for four of these five comparisons indicated that identical or similar ratings were provided for the large majority of patients. The only exception to this rule was the patient–physician comparison for social functioning, showing clear differences between patient and physician scores in almost 40% of the cases. Overall, we conclude that significant others and health care professionals can provide useful information about general aspects of cancer patients' QOL. At the same time, the proportion of exact agreement (about 40% across all comparisons) and the moderate correlations underscore the fact that patient and proxy ratings are frequently not identical.

Head-to-head comparison of the significant others, physicians and nurses as proxy raters of patients' QOL indicated that (dis)-agreement was not consistently associated with the type of proxy respondent. This finding is at odds with some earlier studies among cancer patients (Slevin et al, 1988; Blazeby et al, 1995; Sigurdardottir et al, 1996), in which it has been suggested that health care professionals are particularly poor judges of patients' QOL. Rather, the current findings support our earlier conclusion, based on a careful review of the literature (Sprangers and Aaronson, 1992), that significant others and health care professionals evaluate patients' QOL with a comparable degree of (in)accuracy. Also, given the modest effect of the age, gender and level of education of the proxy raters on the degree of patient–proxy agreement, there is insufficient evidence to prefer one type of proxy respondent over the other.

The level of patient–proxy agreement was dependent, in part, on the patients' performance status. Large discrepancies between patient and proxy ratings occurred most frequently among patients with a slightly or moderately impaired performance status, and less frequently among patients with either a very good or very poor performance status. This finding is in line with an earlier study (Sneeuw et al, 1998), suggesting a U-shaped relationship between patient–proxy concordance and the level of patients' functioning. This pattern can also be understood intuitively, given the smaller potential for score discrepancies in patients with either a very good or very poor functional status. While for such patients the answers to many questions will be evident (i.e. either at the top or bottom end of the scale), ratings are more likely to diverge for patients with an intermediate performance status. This finding is of particular relevance for the possible use of proxy respondents in clinical studies, because it implies that we can rely on proxy-derived QOL information when the need for proxy QOL ratings is most salient. That is, the use of proxy respondents is of particular relevance for those patients who cannot provide QOL ratings themselves due to their poor clinical status.

Interestingly, when comparing responses of the four raters simultaneously, deviant scores of more than one response category appeared to be caused most often by the patients themselves. This might be interpreted as indicating that all proxy raters were unaware of the patients' health experience. A more likely explanation, however, is that the patients' deviant scores reflect responses of questionable validity. For instance, six patients reported having a high level of physical fitness while their significant other, physician, and nurse reported that the patient could carry out only moderate to very light physical activity. On several occasions, such suspect responses by patients were noted by the research assistants. This finding supports the view that discrepancies between patient and proxy ratings should not necessarily be interpreted as evidence of the poor quality of proxy-derived information (Sneeuw et al, 1997*a*, 1997*b*).

The results of this study indicate that judgements made by significant others and professional caregivers about general aspects of cancer patients' QOL are reasonably accurate. The current study does not support an *a priori* preference for significant others over health care providers. One might conjecture that the results would be different if a lengthier, more detailed questionnaire was used. Patient–proxy agreement might be poorer when more detailed information is requested, demanding more precise knowledge of the patients' level of functioning and well-being. Conversely, one could argue that the level of agreement would be increased by more detailed questions, in that the requested information would be more specific and concrete. Additionally, aggregation of several questions in multi-item scales might also lead to higher levels of patient–proxy agreement, given that multi-item scales are theoretically more reliable than single-item measures (Nunnally and Bernstein, 1994). Results pertaining to the U-shaped relationship between patient–proxy concordance and the level of patients' functioning might also be different if a lengthier questionnaire was used. In a study of stroke survivors, which employed patient and proxy ratings on the Sickness Impact Profile, a linear relationship was found, with disagreement increasing with the level of impairment (Sneeuw et al, 1997*c*). This may be related to the fact that scales of this lengthier questionnaire yield many distinctions at the bottom end of the scale.

We conclude that for clinical studies among patient populations at risk of deteriorating self-report capabilities, both patients' significant others and their health care providers can be useful sources of proxy QOL information. At the same time, researchers need to continue to exercise the necessary caution in the analysis and interpretation of their data when using proxy ratings. Additionally, our findings suggest that, in a routine care situation, informal and professional caregivers of cancer patients are reasonably aware of their patients' level of functioning and well-being. Occasionally, however, substantial discrepancies can occur between patient and caregiver QOL judgements. Thus, for optimal patient care, it remains important to verify one's perception by eliciting feedback directly from the patients, whenever possible.

© 1999 Cancer Research Campaign

### ACKNOWLEDGEMENTS

The authors are grateful to the medical and nursing staff of the Department of Internal Medicine of the Netherlands Cancer Institute/Antoni van Leeuwenhoek Hospital, the patients, and their significant others for their cooperation in this study. This study was supported by grants no. NKI 93-139 and NKI 90-A from the Dutch Cancer Society.

### REFERENCES

Aaronson NK (1991) Methodologic issues in assessing the quality of life of cancer patients. *Cancer* **67**: 844–850

Au E, Loprinzi CL, Dhodapkar M, Nelson T, Novotny P, Hammack J and O'Fallon J (1994) Regular use of a verbal pain scale improves the understanding of oncology inpatient pain intensity. *J Clin Oncol* **12**: 2751–2755

Bartko JJ (1966) The intraclass correlation coefficient as a measure of reliability. *Psychol Rep* **19**: 3–11

Blazeby JM, Williams MH, Alderson D and Farndon JR (1995) Observer variation in assessment of quality of life in patients with oesophageal cancer. *Br J Surg* **82**: 1200–1203

Cohen J (1988) *Statistical Power Analysis for the Behavioral Sciences*, 2nd edn, pp. 19–74. Hillsdale, New Yersey: Lawrence Erlbaum Associates

Detmar SB, Aaronson NK (1998) Quality of life assessment in daily clinical oncology practice: a feasibility study. *Eur J Cancer* **34**: 1181–1186

Fleiss JL and Cohen J (1973) The equivalence of weighted kappa and the intraclass correlation coefficient as measures of reliability. *Educ Psychol Meas* **33**: 613–619

Ford S, Fallowfield L and Lewis S (1994) Can oncologists detect distress in their out-patients and how satisfied are they with their performance during bad news consultations? *Br J Cancer* **70**: 767–770

Grassi L, Indelli M, Maltoni M, Falcini F, Fabbri L and Indelli R (1996) Quality of life of homebound patients with advanced cancer: assessments by patients, family members, and oncologists. *J Psychosoc Oncol* **14**: 31–45

Grossman SA, Sheidler VR, Swedeen K, Mucenski J and Piantadosi S (1991) Correlation of patient and caregiver ratings of cancer pain. *J Pain Symptom Manage* **6**: 53–57

Hodgkins M, Albert D and Daltroy L (1985) Comparing patients' and their physicians' assessments of pain. *Pain* **23**: 273–277

King MT (1996) The interpretation of scores from the EORTC quality of life questionnaire QLQ-C30. *Qual Life Res* **5**: 555–567

Kurtz ME, Kurtz JC, Given CC and Given B (1996) Concordance of cancer patient and caregiver symptom reports. *Cancer Pract* **4**: 185–190

Landis JR and Koch GG (1977) The measurement of observer agreement for categorical data. *Biometrics* **33**: 159–174

Lee J, Koh D and Ong CN (1989) Statistical evaluation of agreement between two methods for measuring a quantitative variable. *Comput Biol Med* **19**: 61–70

Macquart-Moulin G, Veins P, Bouscary M-L, Genre D, Resbeut M, Gravis G, Camerlo J, Maraninchi D and Moatti J-P (1997) Discordance between physicians' estimations and breast cancer patients' self-assessments of side-effects of chemotherapy: an issue for quality of care. *Br J Cancer* **76**: 1640–1645

Magaziner J (1992) The use of proxy respondents in health studies of the aged. In: *The Epidemiologic Study of the Elderly*, Wallace RB and Woolson RF (eds), pp. 120–129. Oxford University Press: New York

Magaziner J (1997) Use of proxies to measure health and functional outcomes in effectiveness research in persons with Alzheimer disease and related disorders. *Alzheimer Dis Assoc Disord* **11**: 168–174

Marshall GN, Hays RD and Nicholas R (1994) Evaluating agreement between clinical assessment methods. *Int J Methods Psychiat Res* **4**: 249–257

Medical Research Council Lung Cancer Working Party (1996) Randomised trial of four-drug versus less intensive two-drug chemotherapy in the palliative treatment of patients with small cell lung cancer (SCLC) and poor prognosis. *Br J Cancer* 73: 406–413

Nelson EC, Wasson JH, Kirk JW, Keller A, Clark D, Dietrich A, Stewart A and Zubkoff M (1987) Assessment of function in routine clinical practice: description of the COOP Chart method and preliminary findings. *J Chronic Dis* **40**: 55S–69S

Nelson EC, Landgraf JM, Hays RD, Kirk JW, Wasson JH, Keller A and Zubkoff M (1990) The COOP function charts: a system to measure patient function in physicians' offices. In: *Functional Status Measurement in Primary Care*, Lipkin M (ed), pp. 97–131. Springer-Verlag: New York

Nelson LM, Longstreth WT Jr, Koepsell TD and Van Belle G (1990) Proxy respondents in epidemiologic research. *Epidemiol Rev* **12**: 71–86

Nunnally JC and Bernstein IH (1994) *Psychometric Theory* . McGraw-Hill: New York

Osoba D (1994) Lessons learned from measuring health-related quality of life in oncology. *J Clin Oncol* **12**: 608–616

Osoba D, Rodrigues G, Myles J, Zee B and Pater J (1998) Interpreting the significance of changes in health-related quality of life scores. *J Clin Oncol* **16**: 139–144

Schag CC, Heinrich RL and Ganz PA (1984) Karnofsky performance status revisited: reliability, validity, and guidelines. *J Clin Oncol* **2**: 187–193

Scholten JHG and Van Weel C (1992) *Functional Status Assessment in Family Practice: the Dartmouth-COOP Functional Health Assessment Charts/WONCA*. Meditekst: Lelystad

Schor EL, Lerner DJ and Malspeis S (1995) Physicians' assessment of functional health status and well-being: the patient's perspective. *Arch Intern Med* **155**: 309–314

Sigurdardottir V, Brandberg Y and Sullivan M (1996) Criterion-based validation of the EORTC QLQ-C36 in advanced melanoma: the CIPS questionnaire and proxy raters. *Qual Life Res* **5**: 375–386

Siu AL, Ouslander JG, Osterweil D, Reuben DB and Hays RD (1993) Change in self-reported functioning in older persons entering a residential care facility. *J Clin Epidemiol* **46**: 1093–1101

Slevin ML, Plant H, Lynch D, Drinkwater J and Gregory WM (1988) Who should measure quality of life, the doctor or the patient? *Br J Cancer* **57**: 109–112

Sneeuw KCA, Aaronson NK, Sprangers MAG, Detmar SB, Wever LDV and Schornagel JH (1998) Comparison of patient and proxy EORTC QLQ-C30 ratings in assessing the quality of life of cancer patients. *J Clin Epidemiol* **51**: 617–631

Sneeuw KCA, Aaronson NK, Sprangers MAG, Detmar SB, Wever LDV and Schornagel JH (1997*a*) Value of caregiver ratings in evaluating the quality of life of patients with cancer. *J Clin Oncol* **15**: 1206–1217

Sneeuw KCA, Aaronson NK, Osoba D, Muller MJ, Hsu M-A, Yung WKA, Brada M and Newlands ES (1997*b*) The use of significant others as proxy raters of the quality of life of patients with brain cancer. *Med Care* **35**: 490–506

Sneeuw KCA, Aaronson NK, De Haan RJ and Limburg M (1997*c*) Assessing quality of life after stroke: the value and limitations of proxy ratings. *Stroke* **28**: 1541–1549

Sorensen JB, Klee M, Palshof T and Hansen HH (1993) Performance status assessment in cancer patients: an inter-observer variability study. *Br J Cancer* **67**: 773–775

Sprangers MAG and Aaronson NK (1992) The role of health care providers and significant others in evaluating the quality of life of patients with chronic disease: a review. *J Clin Epidemiol* **45**: 743–760

Stephens RJ, Hopwood P, Girling DJ and Machin D (1997) Randomized trials with quality of life endpoints: are doctors' ratings of patients' physical symptoms interchangeable with patients' self-ratings? *Qual Life Res* **6**: 225–236

Van Weel C (1993) Functional status in primary care: COOP/WONCA charts. *Disabil Rehabil* **15**: 96–101

Van Weel C, König-Zahn C, Touw-Otten FWMM, Van Duijn NP and Meyboom-de Jong B (1995) *Measuring Functional Health Status with the COOP/WONCA Charts: a Manual*. Northern Centre of Health Care Research (NCH): Groningen

Wasson J, Keller A, Rubenstein L, Hays R, Nelson E, Johnson D and The Dartmouth Primary Care COOP Project (1992) Benefits and obstacles of health status assessment in ambulatory settings. The clinician's point of view. *Med Care* 30: MS42–MS49

Zubrod CG, Schneiderman M, Frei E, et al (1960) Appraisal of methods for the study of chemotherapy of cancer in man: comparative therapeutic trial of nitrogen mustard and triethylene thiophosphoramide. *J Chronic Dis* **11**: 7–33

 *© 1999 Cancer Research Campaign*
